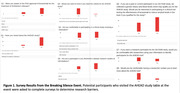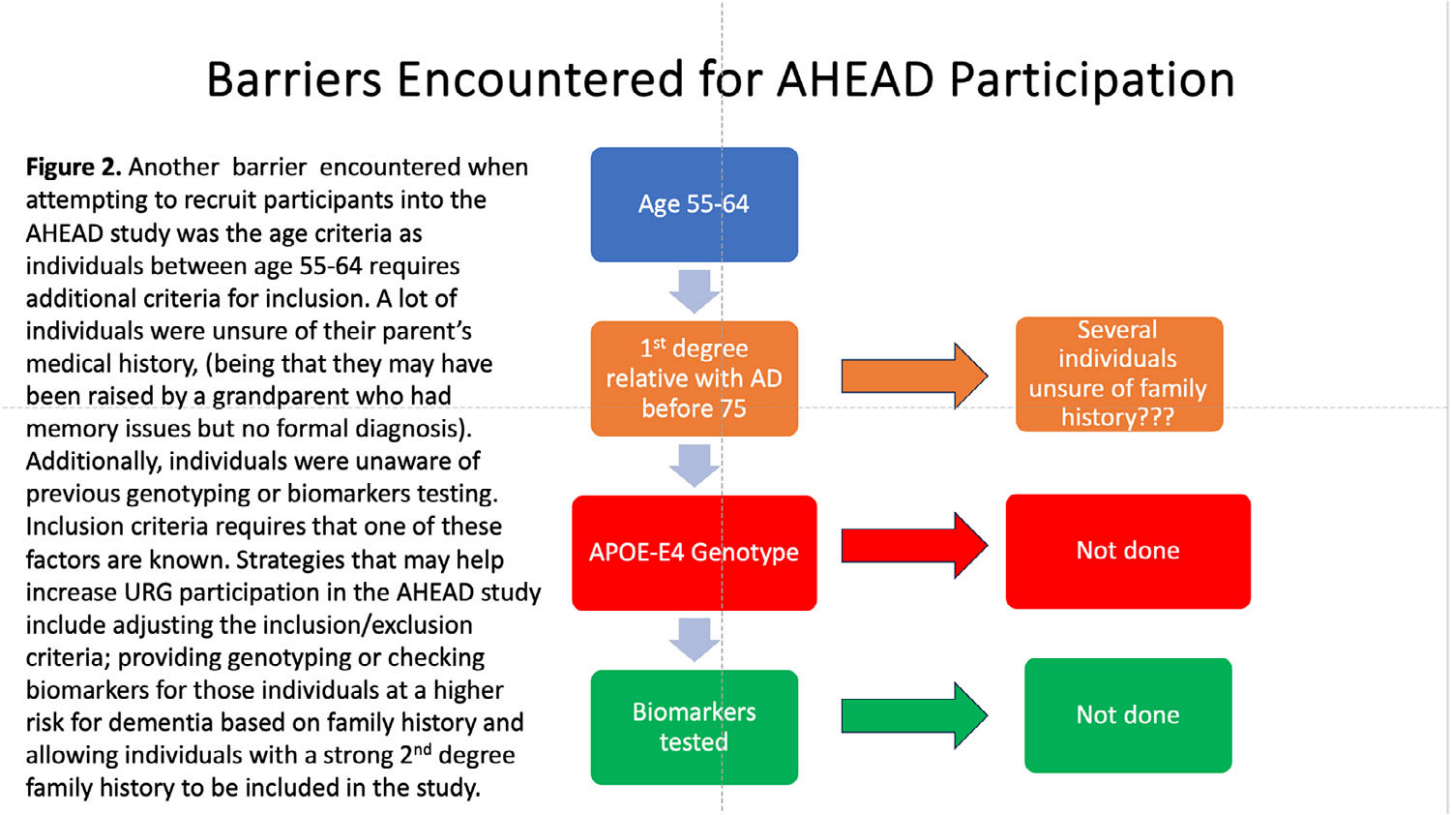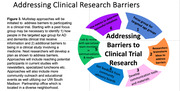# Assessing Clinical Research Barriers to the Participation of Underrepresented Groups in the AHEAD Study

**DOI:** 10.1002/alz.093416

**Published:** 2025-01-09

**Authors:** Taryn T. James, Carey E. Gleason, Diane Carol Gooding, Fabu P. Carter, Cecilia A. Cardenas, Olivia Deering, Aleshia Cole, Kate Cronin, Cynthia M. Carlsson, Shenikqua Bouges

**Affiliations:** ^1^ University of Wisconsin School of Medicine and Public Health, Madison, WI USA; ^2^ Wisconsin Alzheimer’s Disease Research Center, Madison, WI USA; ^3^ Geriatric Research, Education and Clinical Center (GRECC), William S. Middleton Memorial Veterans Hospital, Madison, WI USA; ^4^ Department of Psychology, University of Wisconsin‐Madison, College of Letters & Science, Madison, WI USA; ^5^ Wisconsin Alzheimer’s Disease Research Center, University of Wisconsin School of Medicine and Public Health, Madison, WI USA; ^6^ University of Wisconsin‐Madison, School of Medicine and Public Health, Madison, WI USA

## Abstract

**Background:**

To increase participation of underrepresented groups (URG) into clinical research studies such as the AHEAD study, a study assessing lecanemab in participants with preclinical Alzheimer’s disease (AD), it is necessary to understand and address barriers in an effort to mitigate them. Toward this goal, methods assessing community needs and plans to meet those needs are imperative. Our ultimate goal is to aid URG in understanding the clinical research process and to empower them to participate in AD research so that approved therapies are applicable to them.

**Method:**

This work was made possible by an AHEAD Diversity Recruitment Supplement grant funded to University of Wisconsin‐Madison (UW‐Madison) by the Alzheimer’s Clinical Trial Consortium (ACTC) to increase URG participation in the AHEAD study. Our approaches include using surveys to identify barriers in order to develop a strategic plan to overcome them. Surveys assessed whether potential participants had knowledge of 1) lecanemab, 2) the AHEAD study, and 3) if they would be comfortable in a study involving a medication, among others. Interested individuals were rewarded with a swag bag for completing the surveys

**Result:**

The findings showed that URG were not likely to be aware of lecanemab or the AHEAD study. Additionally, while they wanted to know if they met the criteria for the study, they were less comfortable about a clinical study involving a medicine (Figures 1). Moreover, we identified other barriers to research participation, such as factors that were more likely to prevent URG from meeting study criteria. These included not knowing family history or their AD biomarker status, which was necessary inclusion criteria for individuals age 55‐64 (Figure 2).

**Conclusion:**

Surveys at community events are useful in identifying barriers to the engagement of URG in AD clinical trials. To decrease barriers and push the pendulum towards increased participation of URG in biomedical research, increased educational efforts are needed on AD, the clinical trial process, and drug development. Moreover, researchers must make sure that URG are not ruled out from the study onset, meaning inclusion criteria must consider some unknown challenges such as not knowing medical or family history.